# A story half told: a qualitative study of medical students’ self-directed learning in the clinical setting

**DOI:** 10.1186/s12909-021-02913-3

**Published:** 2021-09-15

**Authors:** Tzu-Hung Liu, Amy M. Sullivan

**Affiliations:** 1 Harvard Medical School, Boston, USA; 2 Family Medicine, Taipei Tzu Chi Hospital, Buddhist Tzu Chi Medical Foundation, New Taipei City, Taiwan; 3 Department of Medical Humanities, School of Medicine, Tzu Chi University, Hualien, Taiwan; 4 Beth Israel Deaconess Medical Center, Shapiro Institute for Education and Research, 333 Brookline Avenue, Room 2ES, MA 02215 Boston, USA

**Keywords:** Self-directed learning, Clinical setting, Medical students, Qualitative interviews, Thematic analysis

## Abstract

**Background:**

Medical educators have promoted self-directed learning (SDL) as an important means of enabling students to take responsibility for their own learning throughout their training and practice. While SDL has been well-studied in classroom settings, it remains a story half told: barriers to and facilitators of SDL in the clinical setting are not yet well described. The goals of this study were to explicate student experiences of SDL in their clinical training and to identify the roles that local social and cultural contexts play in shaping their experiences of SDL.

**Methods:**

To understand students’ conceptualization and experiences of SDL in the clinical setting, we carried out a qualitative study with 15 medical students at Harvard Medical School. The semi-structured interviews were recorded and transcribed. Using an interpretivist approach, data were analyzed both deductively and inductively using the Framework method of content analysis.

**Results:**

Participants described patient care activities as primary motivators for engagement in SDL in the clinical setting. Participants’ descriptions of SDL aligned with Knowles’ steps in SDL, with an additional step of consolidation of learning related to their patients’ diagnosis and management. Participants described using a range of cognitive, social-emotional, and peer learning strategies to enhance their SDL. Participants who described a growth mindset appeared to engage in SDL more easily. Learning environments that fostered SDL were those in which faculty and residents demonstrated an educational orientation, promoted psychological safety, and invited student engagement. Teams with perceived excessive work demands were perceived to be less supportive of SDL.

**Conclusions:**

Our study enhances previous classroom-based models of SDL by providing specific, practical implications for both students and faculty in the clinical training setting. Participants described SDL in the clinical setting as patient-centered, and when effectively implemented, SDL appears to support a mastery rather than performance orientation. Our study paves the way for improving medical students’ clinical SDL and helping them become lifelong learners in the field of medicine.

**Supplementary Information:**

The online version contains supplementary material available at 10.1186/s12909-021-02913-3.

## Background

Since the concept of self-directed learning (SDL) was described by Malcolm Knowles in 1975, medical educators have embraced the principles of this student-centered approach to enable physicians to take responsibility for and drive their ongoing learning [1, 2]. Medical students are expected to cultivate the habits of SDL and develop lifelong learning skills at medical school [3]. As Knowles defined it, “A self-directed learner takes responsibility for their own learning and has internal motivation to develop, implement, and evaluate their approach to learning.” [4, 5] Knowles described SDL as a learning contract between a learner and an instructor and a linear process comprising six major steps: (1) climate setting (creating an atmosphere of mutual respect and support); (2) diagnosing learning needs; (3) formulating learning goals; (4) identifying human and material resources for learning; (5) choosing and implementing appropriate learning strategies; and (6) evaluating learning outcomes [4]. Some criticism of this model includes the primary emphasis placed on individual learners, which, in certain contexts, may be synonymous with a lack of support, and a lack of attention to the social and cultural dimensions of learning [6]. The “Person, Process, Context” (PPC) model, proposed by Hiemstra and Brockett, addresses these potential shortcomings by incorporating necessary social and cultural supports into the SDL model [7–10]. In the PPC model, “Person” refers to characteristics of the individual, such as motivation, resilience, and self-concept, “Process” refers to the teaching-learning transactions such as planning and organizing, and “Context” refers to the environmental and sociopolitical climates, such as learning environment and group culture [10]. Because Knowles’ model and the previous research on SDL focus more on the individual- and process-related dimensions of SDL, this PPC model provides a guiding framework for researchers to address how the contextual dimensions support or hinder SDL [10]..

SDL in the classroom setting, such as problem-based learning in medical schools, has been well-studied in the last two decades [11–14]. Problem-based learning and, more recently, case-based collaborative learning have been developed as tools to help learners apply their learning to clinical cases [12, 15, 16]. These approaches emphasize the development of learner autonomy and intrinsic motivation, with “supported autonomy” or scaffolding provided by the faculty tutor or facilitator guidance [17, 18]. However, with a few exceptions [19], these approaches are systematically implemented primarily in the classroom setting, and the ways in which SDL is experienced and manifested in the considerably less structured clinical environment remain understudied. The complexity, uncertainty, and power differentials in the clinical setting present a very different learning environment compared with the classroom setting, which may pose unique challenges for medical students’ practice of SDL. Previous research suggests that, for novice students, a supportive environment that helps them to navigate these complex clinical learning experiences can improve their SDL [20]. Although the importance of external guidance has been demonstrated to be important for supporting SDL among medical residents [21, 22], similar evidence of how the clinical environment can support or hinder students’ SDL has not been described. These findings align with the “Context” dimension of the PPC model and suggest further studies are needed on medical students’ SDL in the clinical setting.

To further understand how medical students experience SDL and employ SDL strategies in the clinical setting, we carried out a qualitative interview study with medical students in a single medical school in the Northeastern U.S. The goals of this study were (1) to describe student experiences of SDL in their clinical training, and (2) to identify the role that social contexts play in shaping their experiences of SDL. Our overall aim was to determine whether and how students’ descriptions of experiences and social contexts in clinical training could inform and extend current models of SDL.

## Methods

We used an interpretivist paradigm to study students’ experiences of SDL in the clinical setting [23]. This approach embraces a perspective of co-construction of knowledge between researcher and research subjects, and holds that individuals construct their own interpretations and sense of meaning of their experience in the context of their social, temporal, and cultural environments. Because little is known about students’ SDL experiences in the clinical setting, we carried out one-hour-long, in-depth qualitative interviews with students in their clerkship period. We chose interviews for our data collection approach (rather than focus groups) because we wanted to provide individual students the opportunity to describe and explain their experiences in depth. We drew on the Standards for Reporting Qualitative Research (SRQR) and the Consolidated Criteria for Reporting Qualitative Research (COREQ) to guide our analysis and reporting of findings [24, 25]..

In the U.S., medical students typically undergo training that often, but not always, is structured into two years of coursework followed by two years of clinical or “clerkship” training by participating in patient care in hospital and ambulatory settings. At Harvard Medical School (HMS), following preclerkship training, most students enter the traditional clerkship rotation blocks (including medicine, neurology, OB/GYN, pediatrics, primary care, psychiatry, radiology, and surgery) lasting from 4 to 12 weeks in the core clerkships. A few join the longitudinal integrated clerkship (LIC), which extends over a 12-month period and emphasizes continuity with faculty and patients[26]. Both the traditional rotation and the LIC at HMS share the self-study time on schedules, which is intended for reading and SDL. We invited medical students who recently finished their first year of the clerkship in four affiliated hospitals through email invitation to the class. Purposive sampling continued concurrently with data analysis until no new themes were identified and the samples represented the range of student genders, affiliated hospitals, and types of clerkship training. We developed semi-structured interview questions from a review of literature, consultation with local medical education experts, and pre-testing with two other medical students at HMS. The questions in the interview guide (See Supplemental Digital Appendix 1) asked participants to describe their current SDL experiences and their own definition of SDL. Interviews were conducted by one researcher (THL) between May to June 2018 at HMS. All the interviews were audio-recorded and transcribed verbatim, and the transcripts were checked for accuracy against the recordings. All the transcripts were de-identified, and the audio recordings were deleted after the transcription was complete.

We analyzed transcripts using the Framework method of content analysis (See Supplemental Digital Appendix 2for further details) [27]. The Framework method allows for both deductive (pre-defined codes—here, codes representing the elements of SDL and PPC) and inductive (emergent codes—here, either elaboration of the specific, as yet undefined, manifestations of the SDL/PPC components in the clinical setting, or new constructs that do not fit into the deductive categories) approaches [28]. Codes were systematically generated during regular meetings of the two authors (THL and AMS), who read transcripts independently and met to discuss generation and refinement of codes through line-by-line analysis. We created creating a codebook that defined codes and identified inclusion and exclusion criteria, and applied codes to text using Dedoose Software (SocioCultural Research Consultants, CA, USA). We identified, reviewed, and refined emergent themes using a constant comparison approach [29]. Any differences in defining or naming the themes were reviewed and resolved by consensus. We also explored code co-occurrences to identify the relationships between codes. For member checking, we sent interviewees the analytic results and received their comments. Other strategies to promote trustworthiness of the results are shown in the appendix (See Supplemental Digital Appendix 3).

 All methods were carried out in accordance with relevant guidelines and regulations. The Harvard Faculty of Medicine Institutional Review Board (IRB18-0891) approved this study under an exempt status because it presented no more than minimal risk to participants and was determined to be education research with no personally identifiable information. Informed consent was obtained from all the participants.

## Results

We conducted 15 interviews with medical students who recently finished their core clerkships (Table 1). Eleven of the participants were female. The participants were mostly white or Asian. Nearly all participants attended clerkships with traditional blocks, with one participant trained in the LIC. Our analysis found the results aligned with the “Person, Process, Context” model of SDL proposed by Hiemstra and Brockett, with five of the six steps of the Knowles model embedded in the Process dimension. Because the Process element of the Hiemstra and Brockett model overlaps with Knowles’ SDL model, we begin with a description of how our findings align with and elaborate upon these overlapping elements.

**Table 1:** Characteristics of student interviewees (*n* = 15)

	HMS Students
Gender	
Men	4
Women	11
Race/Ethnicity	
Asian	6
Hispanic	1
White	8
Clerkship	
Traditional blocks	14
Longitudinal integrated clerkship	1

### Adapting the SDL model for the clinical setting

When reporting their process of engaging in SDL, participants reported steps that aligned with steps 2 to 6 in Knowles’ linear model. We renamed each corresponding step using the participants’ terms; for example, we restated “defining learning needs” to participants’ language of “identifying gaps in knowledge.” Participants’ descriptions of their SDL process added an additional step of “building a framework in learning,” and this, in turn, was described as leading students to start a new SDL cycle. We thus replaced “climate setting” with “building a framework,” and reshaped the linear model into a circular one.

### The Process Dimension

The process dimension includes six steps of SDL.

#### The Six Steps of SDL

Based on Knowles’ six steps and our analysis of the results, especially from the participants’ own definition of SDL, we identified six steps of SDL in the context of clinical clerkships. When being asked the definition of SDL, the participants referred to a range of steps, usually only three to four per individual, but collectively they referred to all six steps. They described following the steps in this sequence: identifying gaps in knowledge; generating learning topics; finding learning resources; implementing learning strategies; self-assessing learning outcomes; building a framework in learning. The six steps make a continuous cycle of learning and have key components in each step. Among the six steps, finding learning resources was most often mentioned by participants, followed by identifying gaps in knowledge, generating learning topics, and implementing learning strategies (Table 2).

**Table 2:** Emergent themes related to the Process dimension of self-directed learning

Theme: The “Six steps” (a subtheme for example)	Quote
*Step 1: Identifying gaps in knowledge* *(application of knowledge)*	“I feel the gap is that you have to have that knowledge base of what is the classic way that these diseases present, but then you also have to be okay with ambiguity and weighing different competing factors, that would never happen in a UWorld [an online question bank] question.” (Case No.11)
*Step 2: Generating learning topics* *(direct patient care)*	“It wasn’t like ‘Okay, the patient comes in with this, these are the things that you need to research to figure out how to do and what to do for the patient.’ It was more ‘What do you think you need to do for the patient? What do you think are the important diseases or important problems?‘” (Case No.13)
*Step 3: Finding learning resources* *(learning from team members)*	“I feel usually no hesitation with reaching out to whether it’s fellow medical students who I may be on service with, classmates, residents, attendings, or fellows, proposing to them what my thought process is, and then (asking) whether they’ve experienced similar encounters in the past, how they would think about the case, and what they would recommend us as next steps.” (Case No.5)
*Step 4: Implementing learning strategies* *(cognitive strategies)*	“One of the things I like to do is, whenever something comes up on rounds that I don’t understand. … I’ll write it down on a piece of paper, and then I can look it up when I go home. So that’s one way for me to keep tabs on knowledge capturing me.” (Case No.3)
*Step 5: Self-assessing learning outcomes* *(self-reflection)*	“For most rotations, I would track what I’ve done, how many patients I saw or what they had. Like anything important that happened during the day, I would write down. So, I do like to track all the patients that I’ve seen, what conditions they have, what their diagnosis was. That’s a good way to go back and reflect and see what you’ve done.” (Case No.14)
*Step 6: Building the framework in learning* *(big picture for learning)*	“They [The residents] definitely do not help guide you by telling you, ‘Go read this thing.’ … They do not do that. I think the way they help guide you is they might ask you the leading questions about this particular case, to help you recognize this patient’s big picture.” (Case No.12)

#### Step 1: Identifying gaps in knowledge

All student interviewees addressed the importance of detecting their gaps in knowledge, essentially gaps in content knowledge, which mainly referred to knowledge students learned from textbooks or online resources. In addition, about half of the participants described a need to learn how to apply their knowledge to specific clinical cases, and to tolerate the uncertainty and ambiguity that came with “real world” patient cases.

#### Step 2: Generating learning topics

After participants identified their gaps in knowledge, they described generating the learning topics that they could focus on and allocate their limited time and resources. The learning topics were found either by participants themselves or with the guidance of the team members. There were two common pathways that participants went through to generate the learning topics. One was in patient case preparation, in which participants actively collected a patient’s data and systematically presented the case on rounds or teaching sessions. The other path was through direct patient care, when participants were directly involved in primary care and encountered a series of clinical problems.

#### Step 3: Finding learning resources

All student interviewees mentioned that finding learning resources was a critical step in SDL. They used a wide variety of online and printed resources for reading and test preparation. Overall, there were five major types of online resources (ranked from high to low coding frequency): summaries on topics (e.g., UpToDate), question banks (e.g., UWorld), journal articles (e.g., PubMed), visual learning (e.g., YouTube), and direct web search (e.g., Google Search). The first two were used and trusted the most by all the participants. The printed resources named were textbooks, pocket manuals, and question-based books.

 Participants described interacting with people around them to find learning resources. Since medical students were assigned to a team with the intern, resident, and attending physician in each rotation, they naturally and frequently sought help from these people who were more experienced than they. Several participants mentioned that they learned from nurses and paramedics as well. Participants described learning self-directedly by two dominant mechanisms: asking these team members questions and seeking their feedback.

Peer learning and support also played a vital role in promoting SDL. There were three common categories described in peer learning: peer teaching, word of mouth, and shared resources. Medical students can teach and learn from peers and benefit from each other’s experience, knowledge, or ways of approaching a topic.

#### Step 4: Implementing learning strategies

Beyond reading, participants reported a variety of cognitive strategies. There were six cognitive strategies: (1) note-taking; (2) use of question banks; (3) self-quizzing; (4) visual learning (e.g., videos); (5) use of flashcards/mnemonics; (6) teaching to learn/writing study guides. Note-taking, use of question banks, and self-quizzing were the three most commonly described cognitive strategies, while very few participants reported that they learned by teaching others or writing study guides.

The second part of learning strategies in SDL was related to time management. Participants were concerned about efficiency in learning and patient care, just-in-time preparation for rounds or case presentations, and a balance between work and life. Based on the analysis of code co-occurrence, we found participants who reported asking questions and using question banks or question-based books also described better efficiency in learning. For the purpose of just-in-time preparation, participants preferred using the online summaries on topics (e.g., UpToDate).

#### Step 5: Self-assessing learning outcomes

Medical students in the U.S. have shelf exams (standardized exams covering material on one of a range of subjects) and board exams (medical licensure exams). Participants reported spending a great amount of time using question banks not only for preparing for the exams but also for gauging their level of knowledge on certain topics. Participants also regarded feedback seeking as a way of self-assessing their clinical performance. However, only a few participants did this purposefully as a strategy for SDL.

#### Step 6: Building a framework in learning

The sixth step in the SDL cycle was to build the framework in learning. The framework developed throughout the first five steps helped participants identify their knowledge gaps when encountering the next patient case. When participants mentioned the framework in learning, there were two layers of meanings: learning the routine practices or standard operating procedures (SOPs) in the clinical setting, and consolidating their knowledge about a clinical topic or seeing the “big picture” (that is, having a holistic view) of the patient’s diagnosis and management. Participants reported that team members could be most helpful in SDL by helping them integrate their overall clinical understanding of their patients.

### The Person Dimension

We found four themes aligned with the Person dimension of SDL: student motivation, clinical interests, growth mindset, and social-emotional strategies. (Table 3)

**Table 3:** Emergent themes related to the Person dimension of self-directed learning

Themes	Definition	Exemplary Quote
Motivation	Students motivate themselves to provide better patient care and expect themselves to be a well-rounded physician in the future.	“One day when I’m a fellow or an attending that I know that I put forth an honest effort to really lay a good foundation for medical education.” (Case No.4)
Clinical Interests	Students have the freedom to choose whatever clinical topics or specialties they are more interested in and are allowed more time or resources in learning these topics or specialties.	“I think I would give medical students a little bit more power. Some of it [the course] is very scripted. … So, there was a little bit more direction that the students had to be like, ‘I really don’t want to do this, but can I pursue this option for a week?’” (Case No.6)
Growth mindset	Students are willing to work toward the mastery of knowledge or skills, rather than believe their ability is fixed.	“I’ve cultivated it [a growth mindset] through the years. I was not always like this. I think in the first few years of college, I was much more stressed out about grades. And then I hit a point where I realized that no matter how much you’re stressed about it, there are always things outside of your control. … I just went for a different mindset of ‘I’ll try my best.’” (Case No.14)
Social-emotional strategies	Students are able to understand the people and the environment and manage their emotions and social behaviors to achieve their goals.	“I think it’s just about being able to read people. When I’m walking with them [the attendings], on our way across the hospital to see a patient, and they seem to be in a happy mood, there’s a good time to ask a question.” (Case No.1)

#### Motivation

Participants’ primary motivations in learning were to provide better patient care, followed by anticipating how they would work in the future as residents or attendings. Participants who described greater attention to their future doctoring seemed to be more engaged in SDL.

#### Clinical Interests

Some participants expressed a desire for more autonomy to pursue their personal specialty or subspecialty interests, but clerkship requirements often precluded opportunities to explore other interests. About one fourth of the participants said they felt they would more actively pursue SDL if they had greater autonomy in modifying the structure for their own learning purposes.

#### Growth Mindset

About one fourth of the participants reported that a growth mindset was important in facilitating their SDL. Participants who demonstrated a growth mindset focused more on learning itself and expressed fewer concerns about evaluation.

#### Social-Emotional Strategies

Social-emotional strategies were as follows: (1) building relationships with team members; (2) observing team members’ personalities; (3) being aware of situations (e.g., workflow) that might require a change in student behaviors; (4) being sensitive to how they (students) are perceived. These strategies were discussed in depth by almost half of all the student interviewees, and the most commonly used social-emotional strategy was to build relationships with team members. Participants noticed that they had to develop these strategies in their early phase of clinical training so that they could find more learning resources or seek more help from others. They observed team members’ personalities to identify potential resources in terms of asking questions or seeking feedback. They often described being aware of situations, especially when there was a busy workflow, so that they would not ask questions at an inappropriate time. Some of the participants were sensitive to how they were perceived. They cared about their evaluation and did not want to seem low-performing or disengaged.

### The Context Dimension

The contextual dimension refers to the learning climate, culture, and environment that may facilitate or inhibit SDL. Four major themes emerged through our analysis: the learning environment, impression management, group learning culture, and learning activities (Table 4).

**Table 4:** Emergent themes related to the Context dimension of self-directed learning

Theme/Subtheme	Definition	Frequency	Quote
Learning environment			
* Education orientation*	Students gain adequate support from the team members to try, explore, and learn in a clinical setting.	33 excerpts,14 interviews	“I think if I were to be doing something wrong, my attendings and the residents that I worked with have always been very helpful in terms of just either being there and available for questions.” (Case No.10)
* Psychological safety*	Students feel safe to speak up, make mistakes, and learn something they are not familiar with.	30 excerpts,8 interviews	“If you have a good learning environment, they [the residents] really make it so you can feel comfortable just like doing your best, … and then if you’re wrong, it’s completely okay.” (Case No.8)
* Student engagement*	Students can feel engaged when the team members invite them to talk, discuss, or practice.	9 excerpts,6 interviews	“If they extend the invitation like, ‘Do you want to come?‘ Then, that’s also an opportunity to see more.” (Case No.14)
* Opportunities for ownership of patient care*	Students have chances to take the responsibilities for the care of their patients assigned by the team.	10 excerpts,6 interviews	“I feel like I do have more ownership of the patient. And so, in that setting, and because of smaller group team setting, I definitely feel more proactive and wanting to figure out other things.” (Case No.13)
* Sense of urgency*	Students notice the team members just want their jobs quickly done without spare time for teaching.	20 excerpts,11 interviews	“It was drilled into me that people are busy, so don’t ask them questions… It’s just a consequence of the busy system that is there, it should change… That was my biggest struggle.” (Case No.1)
Impression management			
* Looking good*	Students want to perform well, look good, and get a great evaluation which benefits their application for residency.	25 excerpts,11 interviews	“I think there’s always a fear of not looking smart enough… I don’t think any curriculum is really going to be able to help you get over that.” (Case No.4)
* Being constantly * *evaluated*	Students are evaluated almost all the time when they are in the workplace interacting with others.	30 excerpts,14 interviews	“The evaluations of the clerkship are so subjective. It adds a lot of stress. And in some ways, I described to people that it feels like you’re being interviewed every day for a year.” (Case No.2)
* Not seem disinterested*	Students fear that they will get a bad evaluation if they seem disinterested in clinical learning activities.	9 excerpts,5 interviews	“If you’re doing questions, … it looks like you may not be paying attention to what’s going on and that makes you look not engaged. It goes back to that so much of your grade is being part of the team and being engaged.” (Case No.12)
Group learning culture			
* Collaborative learning*	Students are encouraged to collaborate with the team members, learn from them, and contribute to the team’s learning.	15 excerpts,7 interviews	“I think it’s just really important in the medical profession to work closely with colleagues and collaborate and feel comfortable speaking up when there are questions and you’re not sure how to proceed.” (Case No.5)
Learning activities			
* Formal and informal learning activities*	Students can participate in all sorts of learning activities, even if some of them are not mandated in the clinical course.	10 excerpts,7 interviews	“This is actually my advice when I do talk to the younger students, this idea of looking for opportunities outside the formal (activities) like rounds, go see exams, go see tests, go talk to consultants.” (Case No.7)

#### Learning Environment

The learning environment had five subthemes: education orientation, psychological safety, student engagement, opportunities for ownership of patient care, and a sense of urgency. The first four appeared to be facilitating factors of SDL, while the last one was described as an inhibiting factor.

#### Impression Management

 Many participants described a sense of pressure to make a good impression on residents and attending physicians. Participants addressed three topics: looking good, being constantly evaluated, and not seeming disinterested. In a performance-oriented environment, the “fear of not looking smart enough” caused pressure and anxiety among many medical students. In their descriptions of impression management, participants’ SDL appeared inhibited because it took so much attention and energy to perform well rather than learn well. This phenomenon was apparent in our interview group, where every participant talked about the potentially negative effects of being evaluated.

#### Group Learning Culture

 Participants described group learning cultures as varying from department to department, but overall, collaborative learning, such as learning from team members on the ward, was helpful for medical students to engage in SDL.

#### Learning activities

Participants regarded rounds, morning meetings, teaching sessions (lecture or case presentation), and feedback sessions as formal learning activities. Informal learning activities included seminars, workshops, study groups, or any learning activities that were not arranged by the course director. Whatever learning activities the student interviewees participated in, they demonstrated SDL by asking questions and seeking feedback. Beyond utilizing formal learning activities, participants sought informal ones as a way of SDL.

## Discussion

**Fig. 1:**
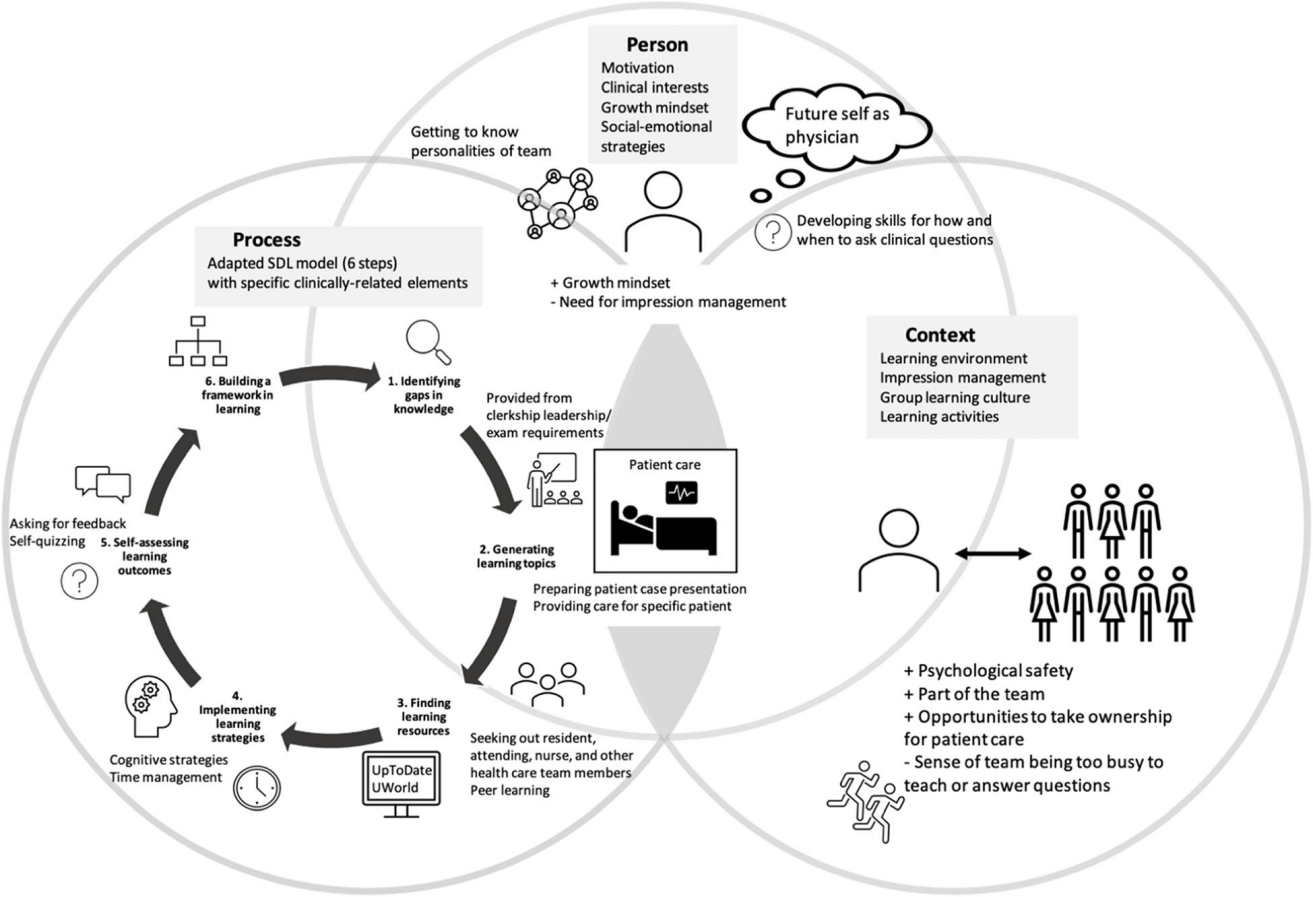
A conceptual model of self-directed learning (SDL) in the clinical setting based on analysis of 15 medical student interviews. Patient care served as the central organizing element for student learning. The six steps shown outside the circle here are drawn from Knowles’ learning model, and the three overarching categories of “Person, Process, and Context” model are drawn from Hiemstra and Brockett. Step 6, “Building a framework,” and specific elements in each step are drawn from student descriptions of SDL

Our findings support the need for a deeper understanding of how, and in what contexts, SDL is experienced and practiced by medical students in the clinical setting. In contrast to the classroom, where the process and context of students’ learning are likely to be both more structured and predictable, the dynamics and complexity of the clinical learning environment call for a more context-specific, nuanced understanding of SDL. Our study suggests that the combined frameworks of Hiemstra and Brockett’s “Person, Process, and Context” model and Knowles’ Self-Directed Learning model provide a useful lens with which to capture this complexity of learning in the clinical setting. The results here, in turn, elaborate upon and extend these models by highlighting students’ experience of SDL in a clinical context. Students’ collective experience of important barriers to and facilitators of SDL in the clinical setting provides potential guidance for both faculty and students in the clinical setting. We summarized and organized these findings into the conceptual model shown in Fig. 1.

One important insight from this study is that patients are both the anchor and the drivers of SDL in the clinical setting, shown in Fig. 1 as the hub of all elements of student learning. Participants described two primary means of identifying their gaps in knowledge and skills: one was in the process of preparing to present a patient’s case, and the other was in their efforts to understand what and how to think about specific patients in their care (Fig. 1, “Process” category). Further, participants who espoused goals of being prepared to provide excellent care for their future patients (that is, when they were practicing more independently) were also those who described the most proactive and well-articulated strategies for advancing their learning (Fig. 1, “Person” category). Contexts that provided more opportunities for ownership of patient care were also reported to facilitate students’ SDL (Fig. 1, “Context” category). Thus, although current definitions of SDL usually emphasize the student-centered nature of the construct, students’ experience of SDL in the clinical setting in this study is primarily patient-centered. This insight can also provide guidance to students and clerkship leaders in developing explicit, well-articulated learning goals and strategies by using patient care as the organizing principle for students’ clinical learning.

The patient-centered focus of students’ learning also leads to a new proposed phase of SDL in the clinical setting that we have described as “building a framework” (Fig. 1, “Process”category, Step 6). Some participants described this as coming to see the “big picture” of the patient’s illness and management. This appears to be a stage in which students consolidate their learning about a specific patient’s illness or disease processes, incorporate multiple perspectives and sources of information related to a patient’s medical and social history, integrate and prioritize information from the patient’s history, physical, and lab results, and understand how to apply content knowledge to direct patient care, often in the face of considerable clinical uncertainty. The elements involved in participants’ descriptions of developing a coherent framework of knowledge and understanding align with three of the six competency domains that have been defined for resident education by the Accreditation Council for Graduate Medical Education (ACGME). These are the competencies of *practice-based learning* (for example, learning to access and evaluate medical information through UpToDate and other learning resources), *medical knowledge* (for example, seeking specific patient-related content knowledge and working to apply that knowledge to evaluation and care of the patient), and *patient care* (for example, learning to identify and prioritize patient care needs). Explicitly recognizing the alignment of this SDL phase with GME competencies could allow for adapting and translating existing tools and guidelines for residents to assist students in identifying and achieving their clinical SDL learning goals. Further guidance and scaffolding from faculty and residents, such as that provided by faculty tutors in problem-based or case-based collaborative learning in the classroom, could enhance this important phase and promote student integration and application of their clinical knowledge.

This study adds to the “Person, Process, Context” model of SDL by describing specific themes that had major influences on medical students’ SDL in each of the three dimensions. Specifically, we found that individual personal characteristics such as clinical interests, growth or performance mindset, and situational/interpersonal awareness were salient in clinical SDL. Providing students with some autonomy to pursue interests in specific fields of training, for example, may facilitate SDL in the clinical setting. A growth mindset, which Dweck first mentioned in the implicit theories of learning, also has strong correlations with students’ learning behaviors [30]. As stated by our interviewees, the growth mindset can be cultivated, and students with this mindset appeared to engage in SDL more easily. Finally, we listed four social-emotional strategies students used in SDL, including building relationships with team members. The examples of social-emotional strategies described by participants also remind us that SDL is about far more than learning on one’s own; SDL also includes learning from team members and peers. This broad array of personal habits, strategies, and mindsets might be guided and enhanced through existing organizing tools such as an individualized learning plan (ILP). Although ILPs have not been used widely in undergraduate medical education, they are used successfully in other educational settings and have some evidence for effectiveness in improving SDL strategies for senior medical students [31, 32]..

In addition to the phase of “building a framework” described above, the Process dimension comprised all other steps of Knowles’ framework except the “setting the climate” step. This is to be expected from the student perspective, as they are unlikely to feel they have the authority to influence the learning climate in their clerkships. The clerkship-specific elements of the SDL process, as shown in Fig. 1, were fairly consistently described by students in this study. These may be useful guidelines for clerkship leaders to share with students to aid them in formulating and achieving their own learning goals.

Finally, the contextual dimension of SDL highlighted factors such as psychological safety and feeling integrated into the team, whereas high stress or high workload environments diminished students’ perceived ability to learn. Clerkship leaders might consider existing tools to enhance psychological safety, such as the CENTRE approach, which provides an explicit process for groups to discuss how to work together with respect and curiosity [33]. In performance-oriented learning environments like medical schools, impression management tactics like image creation and protection have been noted in both the residency and clerkship settings [34, 35]. In our study, the equivalent tactics, looking good and not seeming disinterested, were being used by medical students every day in their training hospitals and was described by some as exhausting.

This study has some limitations. This was a study based on a single institution in the U.S. These may limit the transferability of findings to other institutions. It also includes only the perspectives of students, and future explorations of this topic should include faculty and other team members. In addition, our sample did not fully represent the range of demographic characteristics at HMS, where students in the category of “underrepresented in medicine” make up approximately one fifth of the population; further research should explore whether and how SDL experiences vary among a wider range of student backgrounds. SDL may also vary across specialties and levels of training; however, because we wanted to gain an overall view of SDL in the clinical setting, we did not ask specifically about student experiences in different rotations.

Our study explored medical students’ perception of SDL in the clinical setting and refined the model with the six steps and the three dimensions of SDL. Based on the specific themes of SDL we identified, further studies should be focused on how interventions pertaining to these themes are implemented, and we can examine what benefits in SDL they may bring.

## Conclusions

Our findings suggest that current models of SDL in the classroom setting may be a story only half told. In this qualitative interview study with 15 medical students, our findings contribute to both theoretical and practical understandings of medical students’ SDL in the clinical setting. In contrast to the more student-centered focus of SDL in the classroom setting, participants described SDL in the clinical context as primarily patient-centered. We delineated factors at the personal, process, and contextual levels that students experience as facilitators and barriers to SDL in the clinical setting. These results provide practical guidance to students and clerkship faculty as well as new directions for clinically focused research on SDL.

## Supplementary Information

**Additional file 1:**

**Additional file 2:**

**Additional file 3:**

## Data Availability

The datasets used and/or analysed during the current study are available in an anonymized format from the corresponding author on reasonable request.

## Abbreviations

SDL: Self-Directed Learning
PPC: “Person, Process, Context”
SRQR: Standards for Reporting Qualitative Research
COREQ: Consolidated Criteria for Reporting Qualitative Research
HMS: Harvard Medical School
LIC: Longitudinal Integrated Clerkship
SOPs: Standard Operating Procedures
ACGME: Accreditation Council for Graduate Medical Education
ILP: Individualized Learning Plan
