# Supplementary material for: A story half told: a qualitative study of medical students’ self-directed learning in the clinical setting
Source: BMC Med Educ. 2021 Sep 15;21:494. doi: 10.1186/s12909-021-02913-3 (PMC8444548; doi:10.1186/s12909-021-02913-3)
Supplement: Supplementary file 1 — Additional file 1: [file 12909_2021_2913_MOESM1_ESM.docx]

Supplemental Digital Appendix 1

Interview Guide

1. Please describe for me how you learn an unfamiliar case by yourself recently.

- How do you think about your learning?

2. Can you describe for me a time during your clerkship when you felt you were learning well by yourself?

- What contributed the most?

- Any other strategies in self-learning?

3. Has there been a time when you felt that it was difficult for you to learn the necessary clinical skills by yourself? When was that?

- What would have helped you at that time?

4. Do you prefer to take the initiative in learning if you encounter a new case? Or do you prefer to wait and see? Tell me about it.

5. Here is an example of a self-assessment in self-directed learning. A student was on her internal medicine rotation. She felt a lack of knowledge of some common diseases and used the web-based database for learning. The database also provided MCQs for students to assess their own understandings of every topic. She took the MCQs and was correct on almost all the questions. Therefore, she had a sense that she learned well about those topics.

- How did you get an assessment on your self-learning?

- Can you give me a good example of an assessment? / How about a bad one?

- How did you feel about interacting with those who assessed you?

6. Here is an example of feedback in self-directed learning. A student took care of a patient with urinary tract infection. He was eager to know the empiric antibiotic and its adequate dosage. He looked up the guide to antimicrobial therapy, chose one kind of antibiotics and calculated the dose. He then asked the attending physician to see whether the treatment was appropriate. The attending physician thought he picked the right antibiotic, but the dose was not enough for that patient. The attending physician thus corrected the dosage and taught the student how to adjust the dose according to the patient’s body weight.

- How did you get feedback on your self-learning?

- Can you give me a good example of feedback? / How about a bad one?

- How did you feel about interacting with those who gave feedback to you?

7. Here is an example of supervision in self-directed learning. A student was interested in intravenous catheter insertion. She watched the demonstration video online many times and bore in mind all the steps. She then came to a senior resident in the ward and asked for a chance to perform the intravenous catheter insertion on a patient. The senior resident agreed to supervise her, and she successfully completed the procedure.

- Have you been supervised when you learn a clinical skill by yourself?

- Can you give me a good example of supervision? / How about a bad one?

- How did you feel about interacting with those who supervised you?

8. Please share with me your own definition of self-directed learning.

9. Do you have in mind any specific measure that can promote self-directed learning in core clerkship?

Finally, is there any other thing you want to tell me about your clinical learning experience?
