# Supplementary material for: A story half told: a qualitative study of medical students’ self-directed learning in the clinical setting
Source: BMC Med Educ. 2021 Sep 15;21:494. doi: 10.1186/s12909-021-02913-3 (PMC8444548; doi:10.1186/s12909-021-02913-3)
Supplement: Supplementary file 2 — Additional file 2: [file 12909_2021_2913_MOESM2_ESM.docx]

Supplemental Digital Appendix 2

Phases of Framework Method

| **Phase** | **Description of Implementation** |
| --- | --- |
| Phase 0: Interviewing & Transcribing | The researcher (THL) conducted all the interviews face-to-face with quality audio recording, made the field notes, and proofread the transcripts verbatim. Digital files were uploaded to commercial software. |
| Phase 1: Familiarization with the data | The research team members independently familiarized themselves with four transcripts and made notes in the margin about their thoughts and wrote memos. |
| Phase 2: Generating initial codes/themes | Team members met weekly, read transcripts line-by-line together, discussed comments, potential codes, and the importance of topics. |
| Phase 3: Developing an analytic framework | After coding several transcripts, team members met and compared their open codes and agreed on a set of codes to apply to subsequent transcripts. |
| Phase 4: Applying the analytic framework | Researchers coded transcripts and discussed and reached consensus on codes. Software and spreadsheet were used to assist with analysis. |
| Phase 5: Reviewing codes/themes | The research team examined, defined, and categorized all the codes. Some codes were combined or split for clarifications. Themes were developed from the renewed codes. |
| Phase 6: Charting data into a matrix | All transcripts were reviewed and re-coded. A case chart was created to reduce data and retain meaning from each interviewee. |
| Phase 7: Building the conceptual framework | The research team revisited and cross-checked the themes, provided deeper thoughts of codes, and drew preliminary diagrams to depict relationships among the codes. |
| Phase 8: Producing the report | The research team discussed themes of interest in-depth, wrote thick descriptions of context, and found illustrative quotes. A diagram was developed to capture meanings of the themes thoroughly. |
