# Supplementary material for: A story half told: a qualitative study of medical students’ self-directed learning in the clinical setting
Source: BMC Med Educ. 2021 Sep 15;21:494. doi: 10.1186/s12909-021-02913-3 (PMC8444548; doi:10.1186/s12909-021-02913-3)
Supplement: Supplementary file 3 — Additional file 3: [file 12909_2021_2913_MOESM3_ESM.docx]

Supplemental Digital Appendix 3

Trustworthiness of Analysis

| Criteria of Trustworthiness | Means of Establishing Trustworthiness |
| --- | --- |
| Credibility | 1. Prolonged engagement: in-depth interviews of a sufficiently large, representative sample  2. Researcher triangulation: experienced researchers from different institutes and cultural backgrounds; cross-checking the other team member’ coded transcripts  3. Inter-rater reliability checks in the coding team: high reliability Cohen's kappa (0.82)  4. Peer debriefing on codes and themes with other medical education experts who did SDL studies  5. Member checking: illustrative quotes with interpretation sent to interviewees for their comments |
| Transferability | 1. Thick descriptions of interviewees and contexts  2. Using direct quotes from interviews |
| Dependability | 1. Phases of Framework Method (See Appendix 2)  2. Audit trails: records of raw data, field notes, transcripts, and analytic memos |
| Confirmability | 1. Extensive audit trails: records of raw data, field notes, transcripts, and analytic memos, plus the project proposal, IRB applications and documents, interview guide, codebook, analytic framework, case chart, and manuscript draft  2. Explaining the reasons for theoretical, methodological, and analytical choices throughout the entire study  3. See means to ensure the other criteria of trustworthiness |
